# Bacteriological and clinical profile of Community acquired pneumonia in hospitalized patients

**DOI:** 10.4103/0970-2113.63606

**Published:** 2010

**Authors:** Bashir Ahmed Shah, Gurmeet Singh, Muzafar Ahmed Naik, Ghulam Nabi Dhobi

**Affiliations:** *Department of Medicine, SKIMS, Srinagar, India*

**Keywords:** Blood culture, pneumonia, sputum culture

## Abstract

The aim of our study was to obtain comprehensive insight into the bacteriological and clinical profile of community-acquired pneumonia requiring hospitalization. The patient population consisted of 100 patients admitted with the diagnosis of community-acquired pneumonia (CAP), as defined by British Thoracic society, from December 1998 to Dec 2000, at the Sher- i-Kashmir institute of Medical Sciences Soura, Srinagar, India. Gram negative organisms were the commonest cause (19/29), followed by gram positive (10/29). In 71 cases no etiological cause was obtained. *Pseudomonas aeruginosa* was the commonest pathogen (10/29), followed by *Staphylococcus aureus* (7/29), *Escherichia coli* (6/29), *Klebsiella* spp. (3/29), *Streptococcus pyogenes* (1/29), *Streptococcus pneumoniae* (1/29) and *Acinetobacter* spp. (1/29). Sputum was the most common etiological source of organism isolation (26) followed by blood (6), pleural fluid (3), and pus culture (1). Maximum number of patients presented with cough (99%), fever (95%), tachycardia (92%), pleuritic chest pain (75%), sputum production (65%) and leucocytosis (43%). The commonest predisposing factors were smoking (65%), COPD (57%), structural lung disease (21%), diabetes mellitus (13%), and decreased level of consciousness following seizure (eight per cent) and chronic alcoholism (one per cent). Fourteen patients, of whom, nine were males and five females, died. *Staphylococcus aureus* was the causative organism in four, *Pseudomonas* in two, *Klebsiella* in one, and no organism was isolated in seven cases. The factors predicting mortality at admission were - age over 62 years, history of COPD or smoking, hypotension, altered sensorium, respiratory failure, leucocytosis, and *staphylococcus pneumonia* and undetermined etiology. The overall rate of identification of microbial etiology of community-acquired pneumonia was 29%, which is very low, and if serological tests for *legionella*, *mycoplasma* and viruses are performed the diagnostic yield would definitely be better. This emphasizes the need for further studies (including the serological tests for *Legionella*, *mycoplasma* and viruses) to identify the microbial etiology of CAP.

## INTRODUCTION

Community-acquired pneumonia (CAP) remains a common and serious illness despite the availability of potent new anti-microbials and effective vaccines. In the United States, pneumonia is the sixth leading cause of death from infectious diseases.[1,2] Since pneumonia is not a reportable illness, information about its incidence is based on crude estimates. However, it appears that as many as four million cases of community-acquired pneumonia occur annually and as much as 20% of these require hospitalization.[1] The mortality rate of pneumonia patients in out-patient settings is low, in the range of one to five per cent, but among patients who require admissions to ICU it approaches 25%.[3‐6]

In recent years, both the epidemiology and treatment of pneumonia have undergone changes. Pneumonia is increasingly common among older patients and those with co-morbidity like COPD, DM, renal failure, congestive heart failure, CLD and other conditions.[4] Two major variables that influence the spectrum of etiologic agent and initial approach to therapy are the severity of initial presentation and presence of either co-existing illness or advanced age. Patients with severe community-acquired pneumonia have a distinct epidemiology and a somewhat different distribution of etiologic pathogens than patients with other forms of pneumonia. Similarly, the presence of co- morbidity or advanced age can determine the likely pathogens involved.[7]

Although an etiological diagnosis is optimal in the management of community acquired pneumonia the responsible pathogens are not identified in 50% of the patients even when extensive diagnostic tests are performed.[3,4]

The bacteriological profile of community-acquired pneumonia is different in different countries and changing with time within the same country, probably due to frequent use of antibiotics, changes in environmental pollution, increased awareness of the disease and changes in life expectancy. For instance *Streptococcus pneumoniae* remains the commonest organism leading to community acquired pneumonia in most parts of Europe,[8] United States,[9] UK,[10] Iraq[11] and Delhi.[12] *Klebsiella pneumoniae* is the most common pathogen leading to admission to a medical intensive care unit in Singapore.[13] The problem is much greater in the developing countries where pneumonia is the most common cause of hospital attendance in adults.[14]

In India also the etiological agent of CAP varies with geographical distribution e.g. *Streptococcus pneumoniae* predominates as etiological agent of CAP in Shimla[15] and Delhi[12] whereas *Pseudomonas aeruginosa* pre-dominates as an etiological agent in blood culture positive CAP in Ludhiana.[16]

This study was conducted at Sher-I Kashmir Institute of Medical Sciences (SKIMS) Soura, a tertiary hospital with a heterogeneous population representing patients from almost all parts of the valley in order to identify the etiological profile of CAP hospitalized to a medical ward in Kashmir valley.

## MATERIALS AND METHODS

The study comprised of 100 consecutive samples of blood and sputum of patients over 12 years of age admitted with the diagnosis of CAP to the ward of General medicine SKIMS Soura, Srinagar. CAP was defined as new or progressive pulmonary infiltrates on chest radiograph with at least two of the following four: fever, cough, purulent sputum production or leucocytosis over 10,000/ mm^3^. Patients with radiographic evidence of tuberculosis, pulmonary infarction, AIDS, Leukemia, CCF, Lung cancer and patients on immunosuppressive therapy were excluded from the study. A detailed history regarding presence of fever, cough, purulent sputum production and pleuritic chest pain was noted from the patients at the time of admission. Complete hemogram, renal and liver function tests, chest X-ray P/A view, ABG/electrolytes and fasting blood sugars were done in all patients.

Sputum collection was done at the time of admission for gram staining and AFB staining. Sputum containing more than 25 polymorph nuclear cells and less than 10 epithelial cells per low power field was subjected to gram staining. Sputum was also subjected to bacterial culture on blood agar and MacConkey agar media. In patients who could not expectorate sputum spontaneously, sputum induction was done by three per cent hypertonic saline nebulization. Transthoracic needle aspiration was done in cases where sputum could not be obtained. Two samples for blood culture were drawn from two different sites 30 minutes apart and were inoculated over blood agar and MacConkeys Agar media respectively at 37°C for 24-48 hours.

## RESULTS

The mean age of patients was 53.68 plus/minus14.74 years (range 15-80 years). There were 58 males and 42 females. Forty nine patients were in the sixth and seventh decades of life. Maximum numbers of patients were in the age group 60-69 years (n is equal to 27/100) [Table 1].

**Table 1:** Age and sex distribution of cases

Age (years)	Male	Female	Total
<40	6	12	18
40-49	10	5	15
50-59	12	10	22
60-69	17	10	27
70-79	11	5	16
80 and above	2	0	02
Total	58	42	100

Patients above 40 years of age were more pre-disposed to CAP. The maximum number of patients presented with fever (95%), cough (99%), tachycardia (92%), pleuritic chest pain(75%) and sputum production(65%), and leucocytosis (43%).

Smoking was the most common pre-disposing factor identified in 65% followed by COPD in 57%, Structural lung disease in 21%, DM in 13%, altered consciousness in eight per cent and chronic alcoholism in one per cent. Rates of isolation of organisms were sputum 26/100, blood 6/100, pleural fluid 3/100 and pus 1/100 [Table 2]. The overall establishment of etiological diagnosis was possible only in 29 cases of CAP. The most common organism isolated was *Pseudomonas aeruginosa* (10) followed by *Staphylococcus aureus* (7), *E. coli* (6), *Klebsiella* (3), *Sterptococcus pneumoniae* (1), *Streptococcus pyogenes* (1) and *Acinetobacter* (1).

**Table 2:** Distribution of organisms isolated from blood and sputum culture

	No.	Percentage
Organism from blood culture		
* Pseudomonas aeruginosa*	3	3
*Staphylococcus aureus*	2	2
*Klebsiella*	1	1
Organism cultured from sputum
*Pseudomonas aeruginosa*	9	9
*Staphylococcus aureus*	6	6
*E. coli*	5	5
*Klebsiella*	3	3
*Streptococcus pneumonia*	1	1
*S. pyogenes*	1	1
*Acinetobacter*	1	1

A total of 14 patients, nine males and five females, died. The microbiologic etiology could not be ascertained in seven of the 14 patients who died during hospitalization. The factors predicting mortality at admission were - age over 62 years, history of COPD or smoking, *Staphylococcus pneumoniae*, hypotension, altered sensorium, leucocytosis,and respiratory failure and undetermined etiology.

## DISCUSSION

The microbial diagnosis of CAP was confirmed in 29% of patients with standard sputum and blood cultures. Even with extensive laboratory testing the etiological diagnosis could be confirmed in 47.7% and 75.6% in two north Indian studies viz. Ludhiana[16] and Shimla[15] respectively. The maximum numbers of cases of CAP (67%) were in the more than 50 years age group. This is in accordance to the earlier studies and in community based studies in Finland, the rate of CAP increased for each year of age over 50 years.[17] The most common identified risk factor was smoking (65%), COPD (57%), structural lung disease (21%), diabetes mellitus (13%), altered sensorium (eight per cent), and chronic alcoholism (one per cent). Although it is no different from identified risk factors from India and the West[18,19] there is higher prevalence of smoking in Jammu and Kashmir. It was observed by the global youth tobacco survey Jammu and Kashmir that prevalence of smoking in 13-15 years of school going children was (22.4%) as compared to other northern Indian states (7.1- 16.6%).[20] Also the low prevalence of chronic alcoholism as a predisposing factor for pneumonia can be explained by the fact that there is lower prevalence of chronic alcoholism in the Valley due to cultural and religious factors.

The rate of isolation of organisms from sputum culture and blood culture was 26 and six per cent. Previous Indian studies showed sputum culture positivity in 10-33% of patients[21‐23] which is no different from our observation. Decreased sputum positivity is due to prior use of antibiotics, inappropriate sputum production and non-productive cough. Blood culture positivity of six per cent observed in our study is much lower than observed by others 10-24%.[24,25] This can again be explained by prior use of antibiotics and delay in taking the initial blood culture.

Interestingly, AFB positivity was not observed in our study although AFB has been identified in five per cent cases presenting as acute pneumonia in India[16] and Japan.[26] It can only be explained by the frequent use of floroquinolones as an initial empiric antibiotic therapy in OPD and exclusion of patients with clinical and radiological presentation suggestive of tuberculosis.

The most common organism isolated from sputum culture was *Pseudomonas aeruginosa*, followed by *Staphylococcus aureus, E.coli and Klebsiella pneumoniae. Streptococcus pneumoniae* has been identified as the commonest organism causing CAP all over the world[10‐12,31] but some studies, over the last three decades, have reported higher incidence of gram-negative organisms among culture- positive pneumonias.[27‐31] Most of the patients from whom gram-negative bacteria was isolated were over 50 years of age, smokers or had COPD. It has been reported that old age, smoking and COPD impair pulmonary defenses and pre-dispose to CAP caused by gram-negative bacteria.

The second commonest organism isolated from sputum culture was *staphylococcus aureus*. Two patients were less than 40 years of age and had a preceding virus illness. The high incidence of *staphylococcus* in CAP can be explained by spread of *staphylococcus* from hospital setting to community and *staphylococcus* complicating virus illness esp. influenza.

The overall rate of identification of microbial etiology was 29% which is very low compared to other parts of India: 75.6% in Shimla,[15] 47.7% in Chandigarh,[16] or other parts of world 62% in UK,[10] 68% in Singapore,[13] and 56% in Philippines.[32] This can be explained by the fact that the serology for both atypical and viral pathogens was not done at the time of study.

The mortality rate in our study was 14%. The mortality rate of CAP in various hospital based studies is variable, being 5.7% in a British Thoracic Society multi-centric study[14] to a higher mortality of (21-25%) in other studies.[33,34] Poor prognostic factors at the time of admission were – age over 62 years, altered sensorium, respiratory failure, hypotension, leucocytosis, staphylococcus pneumonia and undetermined microbial etiology. This is in accordance with other studies conducted earlier. However, the etiology remained undetermined in 50% of patients who died during hospitalization. This emphasizes the need of further investigations in patients in whom the bad prognostic factors are present at the time of admission so as to establish the etiology, start early treatment and thereby reducing mortality.

In conclusion gram-negative bacteria pre-dominate in the bacteriologic profile of CAP using conventional sputum and blood culture. The common pre-disposing factors of CAP observed were smoking, COPD, structural lung disease, old age and altered sensorium following seizure. The poor prognostic factors at the time of admission were age 62 years, altered sensorium, hypotension, respiratory failure, staphylococcus pneumonia and undetermined etiology. The clinico-bacteriological profile of CAP in valley is different from rest of India but similar to that observed in northern city of Ludhiana and southern state Karnataka. There is need for further conventional serologic tests for atypical and viral pathogens in all patients admitted with CAP.
